# Central Retinal Artery Occlusion after Rhinoplasty Surgery: A Case Report and Literature Review

**DOI:** 10.1155/2022/9997298

**Published:** 2022-03-16

**Authors:** Mohammad-Reza Ansari-Astaneh, Fereshteh Raoufi, Saeed Shokoohirad, Naser Shoeibi, Mojtaba Abrishami

**Affiliations:** Eye Research Center, Mashhad University of Medical Sciences, Mashhad, Iran

## Abstract

**Aim:**

This study was aimed at reporting a case of central retinal artery occlusion (CRAO) after rhinoplasty. *Case Report*. Unilateral blindness occurred in a 22-year-old woman after rhinoplasty with a history of transient visual loss due to migraine aura and vasospasm. The physical examination of the patient revealed a visual acuity of no light perception in the right eye, a 4^+^ relative afferent pupillary defect, disc swelling, cherry-red spot in the macula, and ischemic retina in the right eye. Based on the diagnosis of CRAO, the patient underwent anterior chamber paracentesis (AC tap) along with treatment with mannitol and intravenous hydrocortisone. Visual acuity improved to 1/10 after a two-month follow-up.

**Conclusion:**

Taking history is very important in rhinoplasty surgery, and vasoconstrictors should be limited in the patients with a suspected history of vasospasm.

## 1. Introduction

Rhinoplasty is a historical surgery mainly conducted for aesthetic issues and functional problems. This surgery is a risky procedure, as the aesthetic outcome is not fully predictable and many acute, chronic, and functional complications have been reported in the literature [1–3]. Orbital and ophthalmic complications are rare. The orbit may be involved through trauma and infection. Swelling may impair lacrimal drainage and subsequently cause purulent dacryocystitis. Moreover, enophthalmos may be developed [1, 2]. Even in an extremely rare condition, blindness may happen. This blindness is mainly due to orbital compartment syndrome, direct trauma to the optic nerve, or central retinal artery occlusion (CRAO). The role of embolization in the case of intranasal injection of local anesthetics should be considered [1, 3]. Herein, we report a case of CRAO in a 22-year-old female one day after rhinoplasty.

## 2. Case Presentation

**Figure 1:**
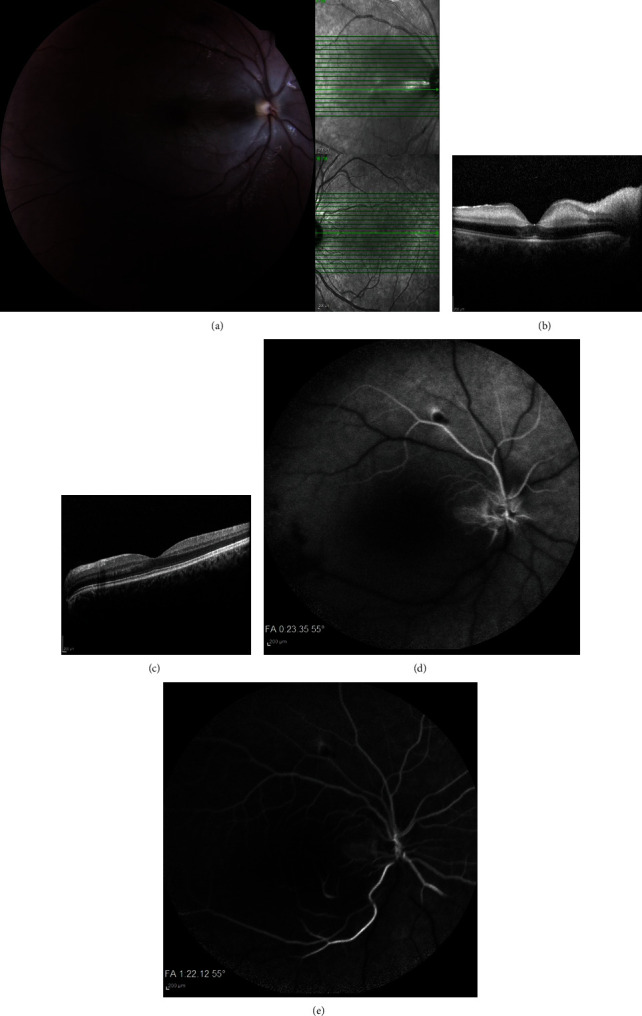
Fundus photography (a), optical coherence tomography (c, d), and fluorescein angiography (f, f) of the patient. Arterial vessel attenuation, cherry-red spot, and edematous nerve fiber layer are seen in the fundus photo (a). Thickening and edema of inner retinal layers in the right eye (b) and normal OCT in the left eye (c).Note: no perfused vessels in FAG still after 0′ 23^″^ (d) and 1′ 23^″^ of dye injection (e).

A 22-year-old woman presented with sudden visual loss the day after rhinoplasty surgery for aesthetic reasons to the emergency room of Khatam Hospital affiliated to Mashhad University of Medical Sciences, Mashhad, Iran. The surgery was conducted under general anesthesia by an ENT surgeon without reported complications during surgery. The surgeon used a topical and local injection of vasoconstrictors (epinephrine) for reducing bleeding and maintaining a good surgical field which is a common practice in nasal surgery. Regarding past medical history, the patient reported intermittently reduced vision since two years ago with a negative cardiologic and neurologic workup. The reason for the transient decrease in vision was stated to be migraine aura due to vasospasm. The visual acuity of the left eye was 20/20, and no light perception was in the right eye. Relative afferent pupillary defect (RAPD) was 4^+^ for the right eye. The anterior segment examination with a slit lamp was normal for both eyes. The posterior segment examination showed nerve fiber layer swelling, cherry-red spot in the macula, vessel attenuation, and ischemic retina in the right eye. It should be mentioned that the left eye was normal. Clinically, the patient was a CRAO case, and the treatment was initiated as soon as possible. Considering this, anterior chamber paracentesis (AC tap) was conducted for the patient. Immediately, 300 cc of 20% serum mannitol infusion was started. Following that, the patient received intravenous 250 mg prednisolone QID and 10,000 units Eprex BID for 3 days. Complementary imaging including optical coherence tomography (OCT), fluorescein angiography (FAG), and fundus photography further confirmed the diagnosis (Figures 1(a)–1(e)).

In order to find the underlying cause of CRAO, different specialists were consulted. All rheumatologic workups were negative, and the nailfold capillaroscopy reported nonspecific morphology abnormalities. Routine laboratory exams, the cardiac system evaluation, and color Doppler ultrasound of carotid vessels were normal. The only positive finding in the studies was the patient's history of transient visual loss with negative workup, which was the reason for the patient's delay in timely visits to the emergency room as the patient experienced vision loss and improvement. The patient received the previously mentioned treatments and was followed for two months. In fact, after AC tap and mannitol therapy, she had a hand motion visual acuity, and a 1/10 visual acuity was achieved at the end of the follow-up.

## 3. Discussion

CRAO is not a common condition in itself; moreover, it may be transient/permanent and happen due to a variety of causes. Among the vast list of etiologies, perirhinoplasty CRAO is an extremely rare condition. The underlying pathology that causes rhinoplasty-related CRAO is not fully investigated. Direct trauma to the orbital apex or compartment-related syndrome is a more common cause in sinus surgeries but not rhinoplasty. Furthermore, in close approaches, such as filler injection, the emboli from filler may cause the obstruction in the central retinal artery as reported by Lin et al. [4].

Local anesthetic and epinephrine injection have been reported as the causes of vasospasm along with hypoperfusion and ischemia for the central retinal artery in the rhinoplasty surgery in the literature. Cheney and Blair [5] reported a case of CRAO in a 37-year-old man who underwent rhinoplasty and experienced decreased visual acuity and pain in the right eye eight hours after the surgery. However, unlike our case, the case of this study had no change in visual acuity even after supportive treatments. They reported that the condition was developed mainly due to the use of epinephrine and vasospasm. Another case of CRAO after rhinoplasty was reported by Alis et al. [1] in a 32-year-old female with complaints of decreased visual acuity in the left eye. A combination of possible etiologies was assumed for this case including direct mechanical trauma, vasospastic/embolic vascular events, and vasospasm by epinephrine.

The literature review revealed that CRAO can also be found after sinus surgeries. Byrd et al. [6] reported a case of CRAO after endoscopic sinus surgery in a 41-year-old female. The patient developed eye pain along with blurry vision in the left eye 12 h after the surgery. Ophthalmology was immediately consulted, and the fundoscopic exam showed pallor of the left retina and optic disk with an inferior visual loss and an afferent pupillary defect. However, after receiving nitroglycerin treatment, the patient had normal-appearing retina and optic disk. They also reported vasoconstriction in the background of epinephrine usage and the presence of autoimmune disease as the main cause of this problem, which was reversed by nitroglycerine. Similarly, Chowdhary et al. [7] discussed a case of CRAO after sinus surgery in a 25-year-old healthy Caucasian male, with painless loss of vision in the left eye. Their case also received hyperbaric oxygen and showed improvement in visual acuity. Fat embolism was proposed to be the cause of CRAO in this patient.

CRAO is still not the only cause of blindness after surgery. It is reported that in an extremely rare condition, a direct injury to the optic nerve or compartment-induced syndrome may be the cause of blindness. However, this usually happens in sinus surgeries, such as ethmoidectomy [8, 9], and has not been reported in rhinoplasty. Monteiro [10] reported another case of blindness related to septoplasty. The cause of blindness was direct trauma to the medial orbital wall, as was evident from the computed tomography scan.

Whatever the cause of CRAO is, the timely diagnosis and treatment affect the prognosis of the patient. The diagnosis mostly relies on the history and physical examination. Cherry-red spots along with the development of retinal opacity are the earliest signs found in fundoscopy. Furthermore, FAG shows a delay or absence of filling in occluded vessels during the arterial phase. OCT can also demonstrate edema in parts of the macula. All these findings were evident in our case in this study.

There are a few things to mention about our patient. First of all, the rhinoplasty surgery has been completed without any complications, and therefore, direct trauma to the optic nerve or compartment syndrome could not be considered the reason. The second point is that all rheumatologic, cardiologic, and neurological workups were negative, and therefore, the systemic problems are not relevant. Third, the issue of embolism cannot be discussed in this patient because no trace of embolization has been observed in the retinal examination, since the use of vasoconstrictors is common in rhinoplasty surgery, and as mentioned in several previous reports, the authors believe that the cause of this rare and severe complication in our patient was related to these vasoconstrictors.

The amount and type of adrenaline used in rhinoplasty surgery vary according to the need of the surgeon, time of surgery, and amount of bleeding from the nasal mucosa. One of the important points in this case that made it unique was the history of transient vision loss in the past, which is important in two ways. First of all, this history has prevented the patient from going to the medical center immediately. The reason was that the patient considered this symptom similar to her previous experiences and expected her vision to improve after a few hours. Second, the cause of reduced vision in migraine, which is often observed in young people, is vasospasm and temporary loss of blood supply to retinal or choroid tissue. The question of whether people with symptoms of migraine are more sensitive to vasoconstriction can logically be raised. Therefore, the authors consider vasospasm to be the cause of this event in this patient and have considered the presence of a history of migraine a predisposing factor although other unknown factors can be considered predisposing factors.

Unfortunately, there is no consensus in the treatment of CRAO; however, many believe that decompression should be made with lowering intraocular pressure using techniques, such as AC tap and infusion of mannitol [11, 12]. Moreover, the use of corticosteroid and vasodilators seems to be efficient in these patients. However, the use of thrombolytic is still controversial and seems not to be related to better prognosis in the CRAO case; nonetheless, it should be used within 6-50 h after CRAO development [13, 14]. Hyperbaric oxygen therapy is another treatment that has been used in the literature; however, further investigation of this treatment is needed.

In the end, it should be mentioned that although CRAO in the background of a rhinoplasty is a very rare disorder, it is a very catastrophic complication for a surgery conducted in case of aesthetic reasons. Moreover, most of the cases that underwent this type of surgery are young people, and this gives higher importance to the condition. Our case in this study developed an acuity of 1/10 at the final follow-up. Surgeons should be more experienced in this type of surgery and have a full vision of the anatomy of the region. Moreover, the use of local anesthetics and vasoconstrictors should be restricted [1]. Our case reported amaurosis fugax attacks before surgery which can predispose the patient for vasospasm attacks and should be considered an important finding, which was not indicated in any of the reviewed cases in the literature.

## 4. Conclusion

In conclusion, CRAO is a rare complication of rhinoplasty. Surgeons should consider and avoid vasospasm and retrograde emboli during rhinoplasty surgery. Taking history is very important, and vasoconstrictors should be limited in the patients with a suspected history of vasospasm. Furthermore, ophthalmologists should keep this etiology in mind, and prompt treatment should be started as soon as possible.

## Data Availability

The FAG and OCT data used to support the findings of this study are available from the corresponding author upon request.
